# Editorial: Liver Fibrosis and MAFLD: From Molecular Aspects to Novel Pharmacological Strategies

**DOI:** 10.3389/fmed.2022.892495

**Published:** 2022-04-28

**Authors:** Ana Sandoval-Rodriguez, Aldo Torre, Juan Armendariz-Borunda

**Affiliations:** ^1^Institute of Molecular Biology in Medicine and Gene Therapy, CUCS, University of Guadalajara, Guadalajara, Mexico; ^2^Instituto Nacional de Ciencias Médicas y Nutrición Salvador Zubirán, Mexico City, Mexico; ^3^Tecnologico de Monterrey, School of Medicine and Health Sciences, Monterrey, Mexico

**Keywords:** MALFD, liver, NASH, NAFLD, steatohepatitis

In this issue, Armendariz-Borunda and collaborators have recruited a group of talented researchers to review important and varied topics in liver fibrosis and recently defined MALFD. This issue of Frontiers in Gastroenterology addressed a range of approaches, basic and experimental to clinical practice, including some up-dated reviews in the field. The issue begins with an article that proposed 10 routine biochemical markers (AGE, ALPK, CHOL, GGT, AFP, APTT, PT, TT, PDW, and PLT) using multinomial logistic regression in a final model of a generic nomogram, covering mild-moderate fibrosis and severe fibrosis, and as stated by the authors, it can be effectively used to predict the degree of liver fibrosis in chronic hepatitis B-infected patients. The predictive value of the generic nomogram for liver fibrosis stage among HBV patients makes it reliable and convenient to use in wide populations (Xu et al.). However, the weakness of the study is that only HVB patients were included, thus the usefulness of the nomogram in other liver fibrosis etiologies must be validated.

It has been more than four decades since the term non-alcoholic fatty liver disease (NAFLD) was coined. NAFLD definition includes three main characteristics: confirmation of hepatic steatosis, non-existence of secondary causes for liver fat accumulation, and no coexisting causes of chronic liver disease (1). However, a great number of studies have emphasized that the disease is associated with metabolic dysregulations, leading an international panel of experts in 2020 to propose a change to the definition and arrive to a new consensus name: MAFLD—metabolic associated fatty liver disease—(2). Controversy regarding the utility of the MAFLD definition was raised; in this issue, the results from Huang et al. of the Third National Health and Nutrition Examination Survey (NHANES III), which included 14,797 participants, demonstrated that MAFLD and NAFLD overlapped, adding to the theory that this new definition miss patients with severe steatosis. Of 12,480 participants, 3,909 were diagnosed with MAFLD and 3,779 with NAFLD; 22.8% of participants were diagnosed with both NAFLD and MAFLD. Ultrasound grading of hepatic steatosis at baseline (1988 to 1994) was linked to mortality information through December 31, 2015, provided by the National Center for Health Statistics (NCHS) of the Centers for Disease Control and Prevention (CDC). In univariable models, data emphasizes MAFLD patients had increased risk for all-cause mortality in a greater magnitude than patients with NAFLD, probably due to metabolic implications (Huang et al.). Race-ethnicity (non-Hispanic white) and presence of hepatic viral infection significantly increased the risks for overall mortality among patients with MAFLD, so these parameters should be taken into account in trials studying the outcome of MAFLD. Efforts from the research and clinical community to sub-phenotype the disease may contribute to the development of new specific treatments.

In the last decades, NAFLD has gained predominance among liver diseases and, as is known, chronic alcohol consumption must be discarded for its diagnosis. However, the effects of modest alcohol consumption on long-term clinical outcomes in NAFLD patients are not definite. Modest alcohol drinking in the study by Wongtrakul et al. was defined as consumption of <21 standard drinks (210 g) per week for men and <14 standard drinks (140 g) per week for women. The meta-analysis suggests that alcohol consumption should be avoided in patients with steatohepatitis or fibrosis (Wongtrakul et al.), since histological follow-up showed that modest alcohol use may diminish the resolution of NASH and increase risk of HCC in NAFLD patients with advanced fibrosis. On the contrary, NAFLD patients with low fibrosis risk may be allowed to engage in modest drinking because they had a lower mortality risk than lifelong abstainers. Data correlates with the cardiometabolic benefits of modest alcohol consumption and with the decreased risk of developing NAFLD in the general population (3–6). However, we should remember that alcohol intake is a risk factor for the development of HCC, both directly via DNA damage from toxic metabolites, oxidative stress, and inflammation and indirectly via chronic liver disease and cirrhosis (7, 8). Furthermore, obesity and DM2 are highly prevalent in the NAFLD population and this synergistic interaction could potentially augment the risk of HCC development (9).

An innovative prospective study conducted in a general Chinese population from 2013 to 2018 showed that 2,452 out of 14,154 participants developed NAFLD, diagnosed by liver ultrasonography. Muscle strength was assessed using a handheld dynamometer to measure HGS (hand grip strength). Hand grip strength was found to be inversely associated with NAFLD (Xia et al.). This result may not come as a surprise as skeletal muscle metabolism can influence insulin resistance and lipid metabolism; however, it is an ingenious way to associate these factors with muscle strength (easier to measure than muscle mass).

Some reviews in this Frontiers in Medicine issue elegantly cover the role of HIF (Hypoxia inducible factors) and epigenetics alterations in NAFLD and NASH development (Holzner and Murray; Rodríguez-Sanabria et al.). These latter reviews remind us of the importance of ROS, inflammation, and metabolic alterations in the development of these hepatic diseases and lead us to keep in mind the new definition of MAFLD. Hypoxia-inducible factor (HIF) are a family of transcription factors that represent a cellular oxygen-sensing system regulating cellular and systemic response to hypoxia (10). Liver hypoxia had been reported in high fat diet fed animals (11), but it remained uncertain what trigger liver hypoxia and HIF activation had in NAFLD. HIF signaling seems to be involved in several key aspects of NAFLD-like steatosis, inflammation, and fibrosis, while HIF2α antagonism in a HFD model of hepatosteatosis had shown promising results (12). In balance, HIF activation appears to be harmful in NAFLD, and may therefore be a useful therapeutic target. Rodríguez-Sanabria et al. describe in detail how DNA methylation processes, histone modifications, and miRNA expression have been closely associated with MAFLD progression. Since epigenetic changes are reversible, and lifestyle and environmental exposure can modify epigenetic patterns throughout life; a variety of epigenetic-based therapeutic interventions seem possible to be developed to modify MAFLD progress or resolution, including dietary microRNAs and supplementation with bioactive dietary compounds such as methyl donors, isothiocyanates, genistein, and resveratrol.

A third review by Qu et al. covers recent new targets and molecules involved in the pathophysiology of NAFLD metabolic dysregulation that could be involved in the progression to liver fibrosis. This review summarizes the therapeutic potential of a variety of molecules implicated in lipid metabolism, inflammation, cell apoptosis, oxidative stress, and extracellular matrix formation (Qu et al.). Such molecules include Fanitol X receptor, Glucagon-like peptide-1, and PPARs agonists, as well as Acetyl-CoA carboxylase, Stearoyl-CoA desaturase, fatty acid synthase, apoptosis signal-regulating kinase 1, and TGF-β-activated kinase 1 inhibitors, among others molecules like Vitamin E and LOXL2 (Lysyl oxidase-like 2) and TGF-β monoclonal antibody in experimental and clinical scenarios of NAFLD/NASH. Although none of the treatments achieved outstanding benefits without significant side effects in a large-scale trial, combinatorial therapies targeting multiple profibrotic pathways could be promising in achieving successful antifibrotic interventions in patients with MAFLD/NAFLD.

Torre et al. stylishly review how the liver immune system orchestrates a response driven by hepatic inflammation that precedes and accompanies fibrogenesis in the liver, where every kind of immune cell and every type of immune response plays a key role in NALFD/MALFD progression. Also, by reviewing therapeutic approaches they aimed to regulate the immune system in NAFLD/MALFD progression and to treat liver fibrogenesis, like CCR2 and CCR5 antagonist, galectin inhibitors, and modulation of macrophage polarization/differentiation (Torre et al.). This Frontiers in Medicine issue continues with a mini review focused on the fact that MALFD/NALFD is the most prevalent liver disorder worldwide and therefore non-invasive strategies for its diagnosis are needed to be developed and widely validated, especially in populations with co-variables like BMI, concomitant diseases, and ethnic background (Segura-Azuara et al.). This article starts defining five hepatic steatosis scoring systems and the reliability and categorization difficulties (Lipid Accumulation Product, NAFLD Liver Fat Score, HS Index, NAFLD Ridge Score, and Fatty Liver Index). Then, it goes on to describe NASH scoring systems and the most used hepatic fibrosis scoring systems so far, including fibromax and APRI. As it is known, liver biopsy remains the gold standard for diagnosis, followed by elastography studies. However, there are contraindications for liver biopsy and elastography requires specialized equipment and technicians; then due to the growing MAFLD pandemic alternatives for screening urge to be available for clinicians, especially for early diagnosis. Our Editorial issue continues by describing the association between cholecystectomy and NAFLD in adults by assessing a cross-sectional study of the National Health and Nutrition Examination Survey in the USA (Xie et al.). Cholecystectomy was found to be positively associated with liver fibrosis and cirrhosis in this population. Gallbladder removal provokes changes in bile flow and concentration of bile acid in the bile duct (13), which may cause chronic cholestasis, NAFLD, and metabolic syndrome (14, 15); through time (<14 years, showed higher incidence of liver fibrosis) these changes act as risk factors. Thus, shunt of bile acids pathway should not be taken as a non-side effect intervention because of the diverse acute complications related to cholecystectomy. Zhang et al. (16) showed that sleeve gastrectomy procedure contributed to significant weight loss and reduced lipids in NAFLD patients and mice model. Molecular mechanisms involved in this effect include increased expression of DUSP1 -a phosphatase with dual specificity for tyrosine and threonine that can dephosphorylate MAP kinase MAPK1/ERK2- and reduce expression of miR-200c-3p. miR-200c-3p is known to regulate the MAPK-dependent signals (p-ERK1/2, p-p38, and p-JNK) that are linked to the promotion of hepatosteatosis via dual-specificity protein phosphatase 1 (DUSP1). This number includes the opinion of Zhang and Yang over T cells' subpopulations during NAFLD-related HCC. It is also described how proliferation of human CD4+ central and effector memory T cells can be affected by high-fat and high-calorie diet in diverse roles in NAFLD development. Likewise, authors end up with the suggestion that T cell manipulation regarding the stage of liver disease and microenvironment may provide a novel approach for HCC treatment, including those related to Gut-Microbiota and miRNA-mediated therapies. This issue explores the relationship between MAFLD and Chronic Kidney Disease (CKD) using Transient Elastography (TE), given the fact that MALFD definition includes metabolic dysfunction and almost all patients with CDK showed metabolic disorders in the form of an atherogenic dyslipidemia. In 335 patients with DM2 and MAFLD, 60.8% had CKD. Patients with CKD had higher mean liver stiffness measurements (LSM) than those without CKD. Surprisingly, steatosis appears to be a better predictor of CKD compared to LSM-assessed hepatic fibrosis (Marc et al.).

As a final point, Fridén et al. designed a study to investigate associations between liver fatty acids measured in three different lipid fractions—cholesteryl esters (CE), phospholipids (PL), and triacylglycerols (TAG)—and liver fibrosis in patients with NAFLD. Also, they wanted to examine whether these associations between liver fatty acids and fibrosis could be confirmed in plasma-derived fatty acids. A positive association between liver PL 22:0 and inverse associations between liver PL 22:6n-3, TAG 18:1n-9, and TAG 18:1 and liver fibrosis were observed. These associations were confirmed in plasma TAG 18:1n-9 and 18:1, however an inverse association was observed for plasma PL 22:0. Total plasma TAG MUFA was inversely associated with liver fibrosis. This result suggests that plasma fatty acids could potentially be used as biomarkers for discriminating patients with NAFLD fibrosis (Fridén et al.).

This issue will engage the reader with an update of MAFLD molecular mechanism and clinics, as well as therapeutic approaches, traveling from oxidative stress, inflammation, fat accumulation and steatohepatitis, through to complications like liver fibrosis, cirrhosis, and hepatocellular carcinoma. This is a journey that could take years in patients, but is addressed far quicker in this issue.

## Author Contributions

AS-R and AT: manuscript writing. JA-B: review and writing of manuscript. All authors contributed to the article and approved the submitted version.

## Funding

This manuscript was supported by the program of Programa de Fortalecimiento de Institutos, Centros y Laboratorios de Investigación 2021 del CUCS.

## Conflict of Interest

The authors declare that the research was conducted in the absence of any commercial or financial relationships that could be construed as a potential conflict of interest.

## Publisher's Note

All claims expressed in this article are solely those of the authors and do not necessarily represent those of their affiliated organizations, or those of the publisher, the editors and the reviewers. Any product that may be evaluated in this article, or claim that may be made by its manufacturer, is not guaranteed or endorsed by the publisher.
